# When more sugar is better—a GPI side chain modification results in a less virulent phenotype during a protozoan infection

**DOI:** 10.1128/mbio.02740-24

**Published:** 2024-11-13

**Authors:** Frank Seeber

**Affiliations:** 1 FG 16: Mycotic and Parasitic Agents and Mycobacteria, Robert Koch-Institut, Berlin, Germany; Albert Einstein College of Medicine, Bronx, New York, USA

**Keywords:** parasitology, *Toxoplasma gondii*, glycobiology

## Abstract

The assembly and function of side chain modifications of glycosylphosphatidylinositol (GPI) units (anchors or free forms) are poorly defined. In a recent study, two enzymes, PIGJ and PIGE, of the protozoan parasite *Toxoplasma gondii* were identified and shown to be involved in the assembly of such GPI side chains (J. A. Alvarez, E. Gas-Pascual, S. Malhi, J. C. Sánchez-Arcila, et al., mBio 15:e00527–24, 2024, https://doi.org/10.1128/mbio.00527-24). PIGJ adds N-acetylgalactosamine to the GPI core structure, while PIGE subsequently adds a terminal glucose. Deletion of PIGJ resulted in the loss of the side chain and, strikingly, increased mortality in infected mice, in contrast to PIGE knockouts. Absence of the side chain led to increased binding of the scavenger receptor CD36 to mutant parasites. In galectin-3 knockout mice, the virulent phenotype of side-chain-deficient parasites was largely lost. While the exact mechanisms remain to be elucidated by more experiments, these findings provide the first evidence for the importance of GPI side chains in parasite-host interactions *in vivo*.

## COMMENTARY

**Fig 1:**
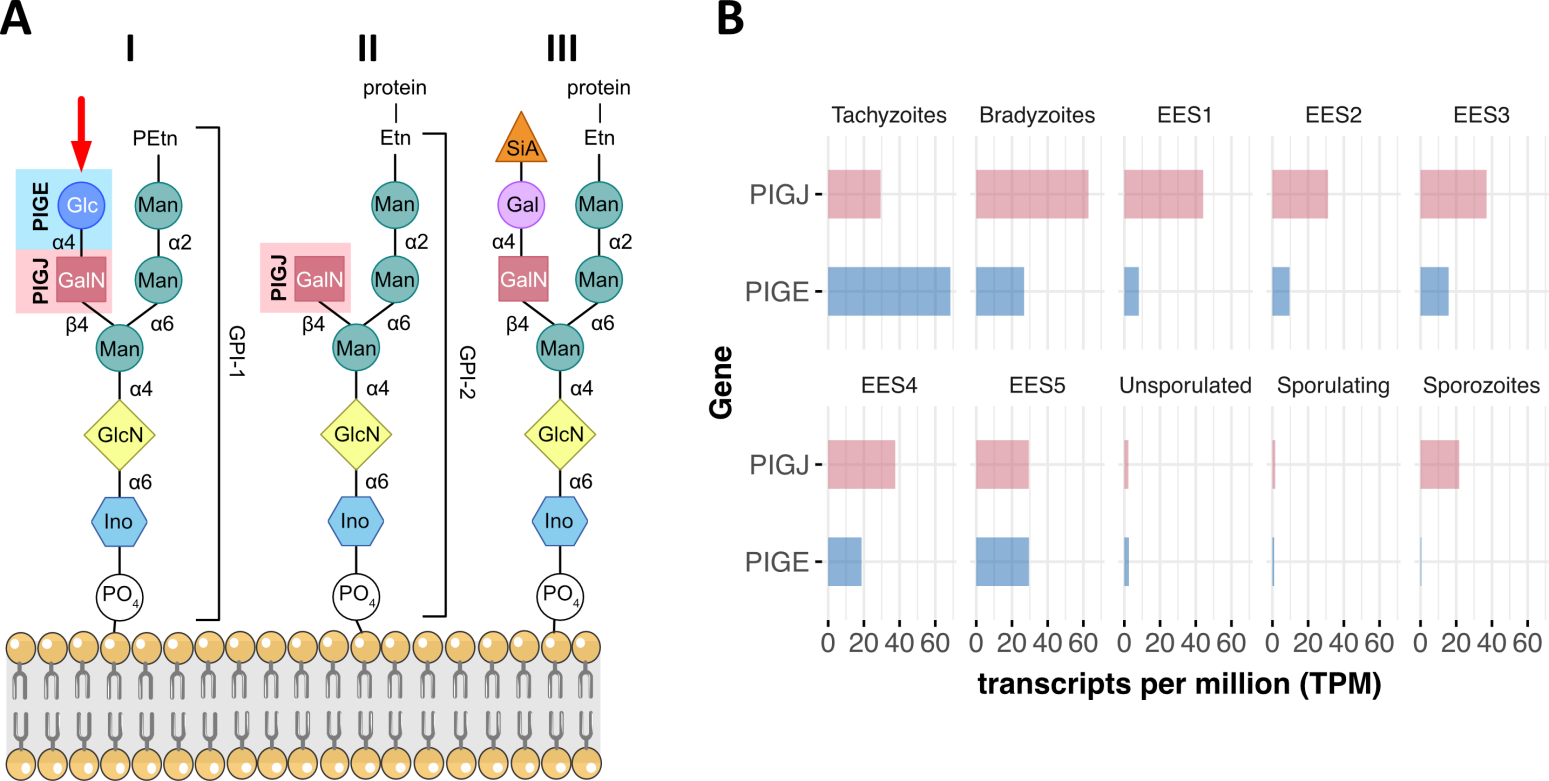
Structural diversity of GPI sidechains and the involved glycosyltransferases in *Toxoplasma gondii*. (**A**) Scheme of protein-free GPI-1 (I), a GPI-anchored protein from *T. gondii* lacking the terminal glucose (II), and, for comparison, a GPI-anchored protein of humans (III) inserted into a membrane via phospholipids. The core structures (base and right “branch”) are identical, whereas the side chain (left “branch”) varies. The glycosyltransferases PIGJ and PIGE in I and II are indicated next to the sugar they incorporate into the side chain. Etn, ethanolamine; Gal, galactose; GalN, N-acetylgalactosamine; Glc, glucose; GlcN, glucosamine; Ino, inositol; Man, mannose; PEtn, phosphoethanolamine; SiA, sialic acid. Arrow points to the terminal glucose in GPI-1 and absent in the GPI-anchored proteins. Modified from reference 4. (**B**) Transcript abundances (expressed in TPM) of PIGJ and PIGE in the different developmental stages of *T. gondii*. EES1-5 are sexual stages within the cat gut. Relevant for infection are tachyzoites, bradyzoites, and sporozoites. The latter originates from sporulating oocysts. Raw data are from reference 5 via ToxoDB (6).

There are certain areas in biochemistry that are less easy to work with than others. One such field is the study of the glycome, with glycosylphosphatidylinositol (GPI) units being one part of it. The complexity of the “sugar code” is huge, and its biological role is, in most cases, ill defined. While the core of the GPI structure is conserved in eukaryotes, the assembly of side chain modifications is not (Fig. 1A). Also, little is known about how they contribute to the function in any cellular system. However, recent developments in mass spectrometry (1) as well as advances in oligosaccharide and glycan synthesis (2) allowed substantial progress to be made. Together with the CRISPR/Cas9 technology for gene knockouts, it is now possible to dissect the synthesis and assembly steps of these complex entities also in protozoan parasites (3).

Recently, in *mBio*, Alvarez et al. (7) reported new insights on the enzymes that transfer side chains onto the GPI anchor of the protozoan parasite *Toxoplasma gondii*. The role of these side chains has so far only been studied in higher eukaryotes, which pointed to important roles in multicellular organisms (8). However, what consequences their loss might have not only on the parasite itself but also on the pathogen-host interaction has not been studied so far in any system.

Results from a previous genome-wide knockout screen in *T. gondii* (9) suggested that all enzymes required for assembly of the core GPI structure would be essential *in vitro* (3), making it difficult to work with but also less informative since dead parasites do not tell us much. Consequently, the authors focused on two potential glycosyltransferase genes, subsequently called PIGJ and PIGE, that had shown little fitness defects in that earlier *in vitro* screen and which were now confirmed by generating individual gene knockouts. Through a series of comprehensive state-of-the-art MS experiments of the knockouts in comparison to wild-type (wt) parasites, Alvarez et al. (7) found that PIGJ adds N-acetylgalactosamine (GalNAc) subsequently to the assembly of the tri-mannose core structure, at the first mannose (Fig. 1A). Similar experiments for PIGE show that it is responsible for adding the terminal glucose to GalNAc, confirming the previously described Glc-GalNAc sidechain structure of *T. gondii*’s GPI anchors (10, 11). But do these modifications have any effect during the course of infection with *T. gondii* tachyzoites?

To provide some answers, Alvarez et al. (7) had to go through a large body of work. To begin with, they infected two different strains of lab mice with ∆*pigj* and ∆*pige* tachyzoites of a strain that usually causes no mortality. Strikingly, ∆*pigj* infection caused death in 60% of animals, while wild-type and ∆*pige* parasites showed zero mortality. When animals previously infected with wt tachyzoites were challenged with a strain lethal for lab mice (“virulent” strain), this did not cause death (as expected due to the formation of protective immunity). However, such secondary infection with ∆*pigj* caused again a similar degree of mortality, which was not seen with ∆*pige* parasites. While some experimental details (e.g., also analyzing the complemented strain *in vivo*) need future attention, it is evident that tachyzoites with a deletion in the GPI side chain cause lethal outcomes during primary and secondary infections. However, the terminal glucose is not involved in whatever the mechanism for this higher virulence is. What could be the reason for this unexpected phenotype?

Alvarez et al. (7) performed many experiments and ruled out some of the usual suspects, such as the induction of inflammatory cytokines like TNFα, IFNα, and IFNβ1; differences in complement C3 binding to the parasite’s surface; or differences in effector functions of sera from chronically infected immune mice. Moreover, GPI-anchored surface proteins were unaffected and still surface bound. Based on these and several other experiments, the authors conclude that for many of the known immune mechanisms being able to control acute infection, the GPI side chain in the PIGJ mutant seems not to play a decisive role. What else then could be the reason for the increased virulence of the ∆*pigj* strain?

While no definitive answer was provided by Alvarez et al. (7), some tropism for macrophages was observed, mediated, in part, by the scavenger receptor CD36. Absence of the side chain resulted in increased binding of recombinant CD36 to mutant parasites. Moreover, galectin-3 (gal-3), previously reported to bind free GPI from *T. gondii* (12), had a significant influence on virulence. When gal-3-deficient mice were infected with either wt or ∆*pigj* tachyzoites, the virulent phenotype of the latter in terms of host death could no longer be observed, although some parameters of infection severity were still present. This indicates that binding of the side chain to gal-3 is responsible to some extent for the severity of acute infection. While Alvarez et al. (7) discuss various roles of CD36 and gal-3 and their possible consequences during infection in this context, I would like to highlight two additional aspects that might also be relevant.

Infection of mice with tachyzoites by the intraperitoneal route (as used by Alvarez et al. [7]) is common laboratory practice. However, it is known that the route of infection and also the parasite stage have a profound effect on infection outcome and the immune responses it provokes when compared to the oral route with bradyzoites-containing tissue cysts (13–15). Moreover, mice are no omnivores, meaning that they rarely, if at all, ingest tissue cyst-containing meat. Rather, it is the oral uptake of the environmental stage (oocysts/sporozoites) that usually leads to infection in nature (16). Consequently, it would be very interesting to see whether the virulence phenotypes seen with the ∆*pigj* and ∆*pige* tachyzoites will be the same when mice are infected orally with bradyzoites/tissue cysts, or even better with oocysts. Transcriptomic data from the literature point to some interesting differences in the abundance of PIGJ and PIGE transcripts in the sexual stages and oocysts (Fig. 1B). Unfortunately, infection with oocysts/sporozoites would require the prior generation of transgenic oocysts in cats, something that only a few labs can do. However, in the not-so-far future, *in vitro*-generated oocysts might be available for this purpose (17).

Galectin-3 has been reported to facilitate the recruitment of IFNγ-inducible guanylate-binding proteins (GBPs) to damaged or otherwise foreign-marked pathogen-containing intracellular vacuoles (18). Interestingly, a similar mechanism has recently been suggested for galectin-9 (gal-9) and GBP2 in *T. gondii*-infected cells. In a report awaiting peer review, Kravets et al. (19) found gal-9 to co-precipitate with GBP2 from IFNγ-stimulated infected murine cells. Replication of type II tachyzoites was observed to be increased in ∆gal-9 mouse fibroblasts 24 h post-infection. Whether gal-9 plays a protective role during mouse infection was not evaluated in this study. Given the ubiquitous distribution of gal-3 and its reported involvement in first-line defenses during many infections (20), it is tempting to speculate that gal-3 might also play a similar role in marking tachyzoites-containing vacuoles and thus contribute to their elimination.

In essence, the observation that the GPI side chain in *T. gondii* reduces the apparent virulence-enhancing effect of the core GPI structure when presented unmasked to the mouse immune system provokes many more studies, not only in this parasite-host relationship.
